# The First Treatment for PKU: The Pioneers—Birmingham 1951

**DOI:** 10.3390/ijns7010019

**Published:** 2021-03-20

**Authors:** Anne Green

**Affiliations:** Birmingham Children’s Hospital, Steelhouse Lane, Birmingham B4 6NH, UK; agreen4168@aol.com

**Keywords:** phenylketonuria, screening, dietary treatment, Birmingham history

## Abstract

Prior to the introduction of newborn screening, Phenylketonuria (PKU) was a devastating disorder with affected individuals usually committed to a life in care in large institutions (asylums). Newborn screening only began after it was shown that those with PKU could be treated with a modified diet and could subsequently lead normal lives. The first production of a diet and the demonstration of its effectiveness was thus a key milestone in the history of both PKU and newborn screening, and took place in Birmingham, UK, in 1951. The pioneers were a two-year-old girl called Sheila Jones, her mother Mary, and three dedicated professionals at Birmingham Children’s Hospital: Evelyn Hickmans, John Gerrard and Horst Bickel. Together, they changed the course of PKU for those across the world. This review summarises the history and achievements of this team who opened the door to PKU treatment and the introduction of newborn screening.

## 1. Introduction

Phenylketonuria (PKU) is a rare, inherited defect in the metabolism of the amino acid phenylalanine, first described in 1934 [1,2]. Until 1951, a diagnosis of PKU offered a bleak future with no options for treatment. Most affected individuals eventually presented with severe learning disabilities (previously referred to as mental retardation or mental defectiveness), and required long term, institutionalised care. Many spent their lives in asylums from their earliest years and in the 1930s and 1940s, diagnoses of individuals, with what became known as PKU, were identified by testing adults resident in these institutions. It was estimated that the incidence of PKU in the general population in the UK was about one in 50,000 [3], and that there were some 1600 affected individuals so institutionalised in Great Britain at that time [4]. During these early days however, many cases remained undiagnosed. By the early 1950s, a few infants with learning disabilities were detected and some younger siblings of known cases were also diagnosed. Since the 1960s, it has been possible to identify individuals with PKU by whole-population screening of newborn infants, thereby enabling their treatment using a low-phenylalanine diet at a very early age. A truly life-changing prognosis for affected individuals occurred as a result of implementing this practice.

The remarkable transformation in the life opportunities for those with PKU only became real when it was shown that a specially formulated diet could be produced and that it had a beneficial effect on their life chances. The demonstration of this possibility occurred at Birmingham Children’s Hospital in 1951. The idea of a special diet for the treatment of PKU was not new, and of particular importance in the history is the contribution made by Dr. Louis Woolf. The ideas he shared freely with the Birmingham team in their development of the first diet have been recently documented in publications to mark his 100th birthday [5,6,7], and with his help, a prototype diet was attempted in Birmingham for the treatment of a two-year-old girl, Sheila Jones. The three professionals directly involved in the manufacture and application of the diet were pioneers, without whom the story of PKU would have been very different. Without their work, effective treatment for PKU would have been delayed, and the necessary stimulus for the early introduction of population-wide newborn screening would also not have occurred so rapidly. This would have been to the detriment of those not only with PKU but for those with other conditions subsequently included in newborn screening programmes.

The detailed scientific findings of this key work in Birmingham were published in 1954 [8,9] and Sheila’s name has become firmly established in the medical history of PKU. However, it is also important, in this Special Issue reviewing the history of newborn screening, to remind ourselves that scientific/medical advances have a human dimension. In remembering the scientific triumphs of the first case of dietary treatment for PKU in Birmingham in 1951, seventy years ago at the time of writing, we should also think about Sheila’s family. Sheila was lost to follow-up in the health care system and her mother, Mary, never knew how important her own role was in unlocking a future for others with PKU, when she persisted in demanding treatment for her daughter. Sheila’s family story has now been published [10], and it can only be concluded that the family too were pioneers and part of the team who changed the course of PKU across the world, which culminated in the introduction of newborn screening.

## 2. Mary

Mary Rooney was born in 1917 and grew up in a farming community in the northwest of Ireland. The economic circumstance in Ireland at that time provided no paid work in rural areas, and so Mary left home to find work. World War II created a need for labour in England, and Mary arrived in Birmingham in 1942/3, a large, industrialised city of approximately 1 million people. This was a very different environment for her as she secured a job at a large car works in South Birmingham, redeveloped for aircraft manufacturing as part of the war effort. She initially lived on her own in rented accommodation, and was to know poverty all her life [10].

Mary married Edgar James Jones in May 1945 and had a son, Terry, in 1945 and a second son, Trevor, in 1948. Sadly, the marriage broke down. Mary had no contact with her family in Ireland, and no longer had her job at the car works. She moved with her two sons to a modest ground floor flat with two bedrooms in Matlock Road, Tyseley, in the southeast of Birmingham. Social care in the community was rudimentary and letting the main bedroom to two lodgers provided her with some income. Sheila was born in 1949, and a third son, Philip, in 1954.

**Figure 1:**
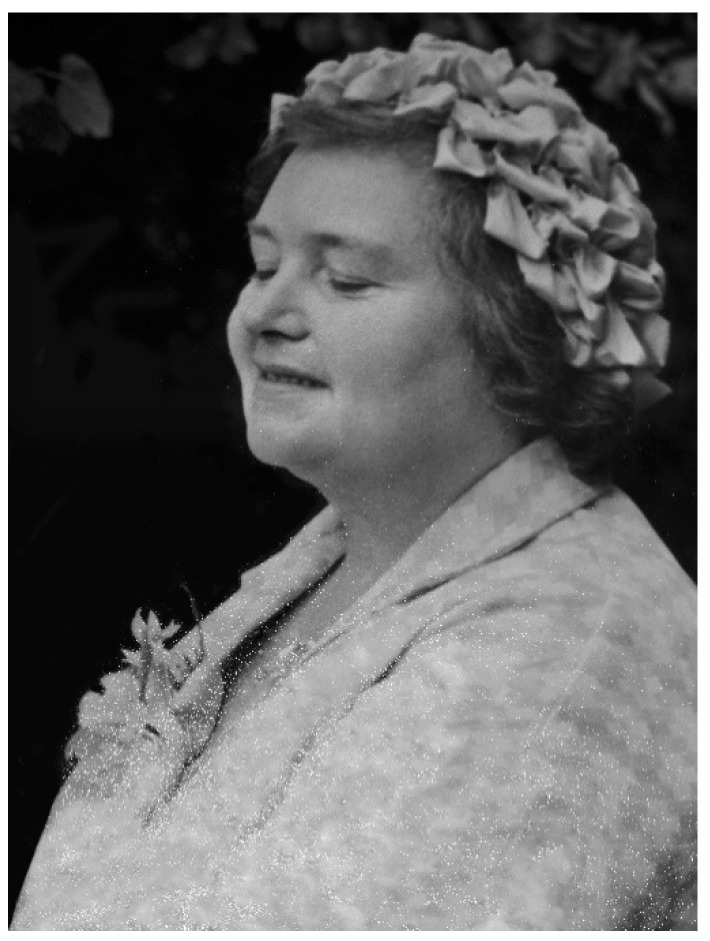
Mary Jones (1917–1981) in 1971 (photograph reproduced with kind permission of the Jones family).

For reasons not known, the family moved to Aston, a severely deprived area north of the city in 1956. Extreme poverty, a child with learning disability, three other children, one-bedroomed back-to-back living [10] (pp. 62, 124) [11,12], no family support and limited social services, together resulted in a deterioration of Mary’s health. She looked after her family for two years under these circumstances before finally succumbing to a complete mental breakdown. She was admitted as a short term, voluntary patient to Highcroft Hospital [10] (p. 64), [13] a local psychiatric unit, on two occasions in 1958/1959. She was pregnant on her second admission and had a fourth son, Liam. Sheila was admitted to Chelmsley Hospital, a hospital for mental deficiency using the terminology of the day, while her mother was in Highcroft, and her brothers spent unhappy times in two different local children’s homes. Upon her recovery, Mary, with her family, continued to live in Aston until the back-to-back houses were demolished in the 1960s (Figure 1). They then moved to North Birmingham, but Mary became unwell during the late 1970s, and died aged 64 years in 1981, from cancer.

## 3. Birmingham Children’s Hospital, 1951

**Figure 2:**
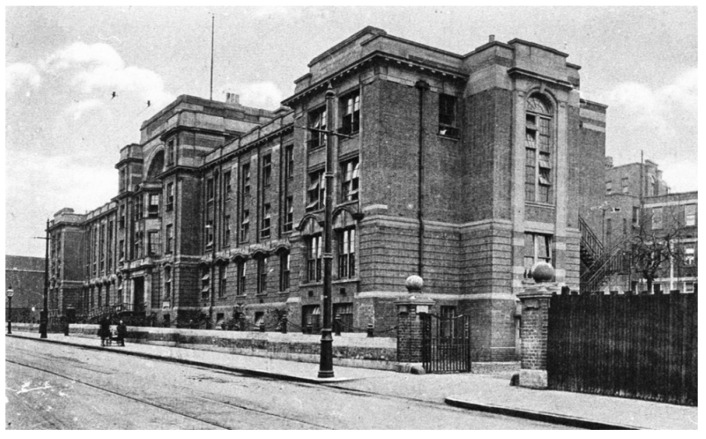
Birmingham Children’s Hospital circa 1950s.

**Figure 3:**
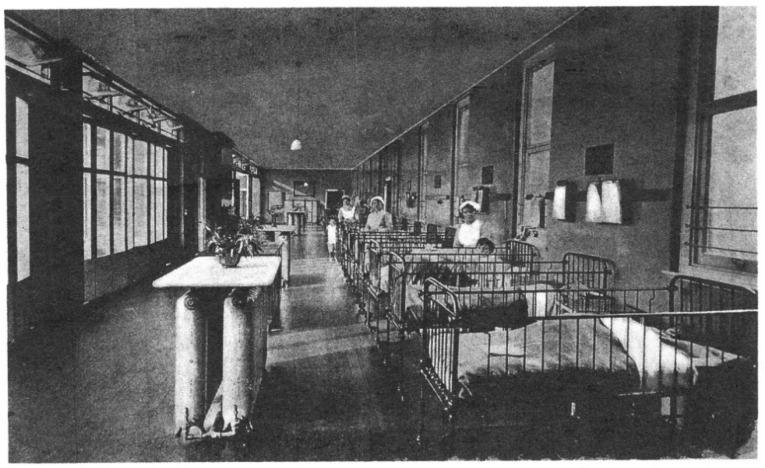
Ward at Birmingham Children’s Hospital circa 1950.

Sheila’s diagnosis and treatment would not have been possible had not three dedicated professionals been working at Birmingham Children’s Hospital (BCH) in 1951 (Figure 2): Evelyn Hickmans, John Gerrard and Horst Bickel. The hospital had opened in 1917 and by 1951, it had 300 beds (Figure 3) and was a focus for new ideas and increasing specialisation in scientific medicine [14]. A key figure throughout this development was one of the early medical pioneers of Paediatrics, Leonard Gregory Parsons (1879–1950) [15,16] as the first Professor of Child Health (1928–1946) at the University of Birmingham. When Professor Parsons retired in 1946, James Maclure Smellie (1893–1961) was appointed [17] and it was under the clinical leadership of these two men that Birmingham Children’s Hospital became a specialist centre where clinical investigations based on laboratory testing occurred. This approach had led to the establishment of a biochemical laboratory, and the appointment of Evelyn Hickmans (1882–1972) as the first biochemist for the hospital in 1923 [18,19,20]. By the late 1940s, Dr. Hickmans had expanded the biochemistry department to provide the facilities and expertise necessary for Sheila’s diagnosis and treatment. She developed microanalyses for use in young children, and introduced paper chromatography, then in its infancy, for the study of amino acids.

The close relationship between science and medicine fostered during this period attracted John Gerrard (1916–2013) to work at Birmingham circa 1945. Following his service in WWII as a Medical Officer, he completed his training in paediatrics under Professor Smellie and became a consultant in 1951 [21]. Part of his duties at BCH was to run a special clinic for children with learning disabilities (in those days referred to as mental retardation), a developing area at the time. Attracted to Birmingham at this time also was a young doctor from Germany, Horst Bickel (1918–2000), who arrived in 1949 to undertake a Ph.D. in Dr. Hickman’s laboratory, in the field of amino acid disorders [22]. He remarked on his arrival, “*I was surprised by the high standards of Paediatrics in this English hospital”* [23]. This was the clinical environment which enabled the pioneering work that led to the first dietary treatment for PKU.

## 4. Sheila—Diagnosis

**Figure 4:**
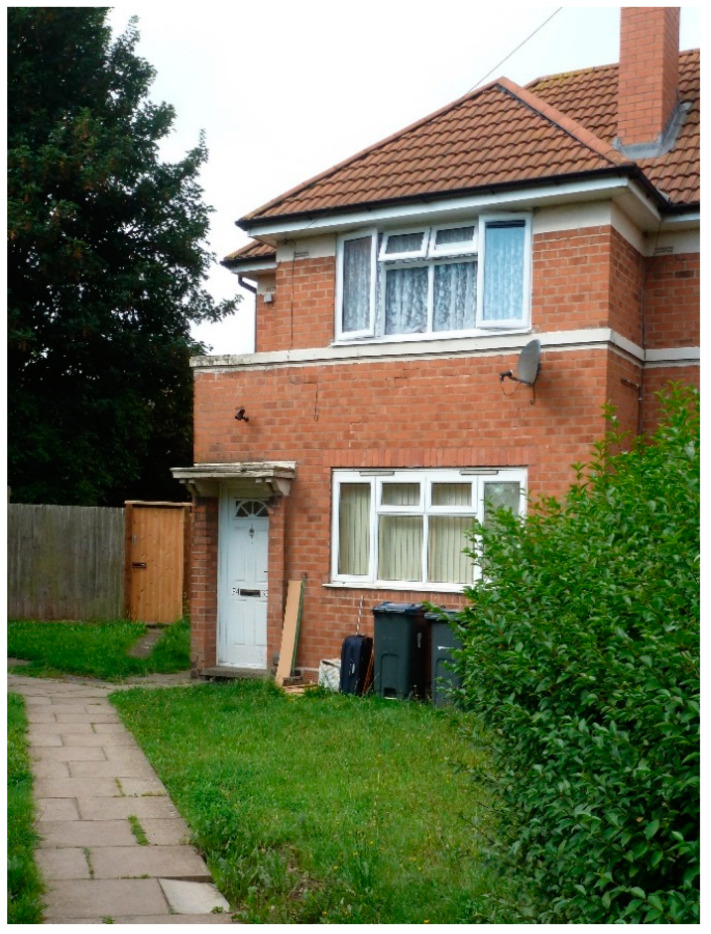
Sheila’s home in Matlock Road (ground floor flat), Tyseley Birmingham 1949–1956 (photograph taken 2019).

Sheila was born normal at term on 2 October 1949 and went home with her mother to live at Matlock Road with her two brothers (Figure 4). She was a strong, healthy and well-nourished baby.

It soon became clear to Mary that Sheila was not developing as she should; she had not started to sit until about nine-months-old and was behind in other aspects of her development. Mary took Sheila to their family doctor at around one year of age, and he referred her to Birmingham Children’s Hospital for an opinion.

Sheila was seen at 17 months of age in Dr. Gerrard’s clinic on 13 March 1951. She had been well looked after by her mother and was well nourished. She had no abnormal physical signs, apart from rough skin with some eczema on her face, but she could not stand or walk and had no speech. She lay in her cot crying and groaning, rocking and continuously rolling her head from side to side and banging it on the pillow. She had fair hair and had an unusual mouse-like smell.

Dr. Bickel had asked Dr. Gerrard: “*Why do you not test the retarded patients for PKU?*” and had introduced the ferric chloride testing of urine from patients at the clinic [23]. Sheila’s urine was tested some weeks later, on 14 April, and was only the third patient they had tested—this would be their first case. The doctors sent a letter to the family doctor, saying “*Sheila suffers from phenylpyruvic oligophrenia. As far as we know no line of treatment is of any avail, but we will admit her to hospital in a month or two for further investigation”.* Sheila continued to live at home until her hospital admission in September 1951, when she was almost two-years-old [9,10] (p. 35).

Analysis in Dr. Hickmans’ laboratory found large quantities of phenylalanine in Sheila’s blood and urine; plasma phenylalanine was approximately 40 times normal with a corresponding low tyrosine level, about half normal. These results combined with increased phenylpyruvic acid in her urine confirmed the diagnosis of PKU. Blood and urine from Sheila’s two brothers and mother showed no abnormalities.

Dr. Bickel recorded: *“The mother was in despair and could not share our excitement about the rare diagnosis, nor our interest in the strong phenylalanine spot on the chromatogram. Instead, she waited for me every morning before the laboratory door, making quite clear it was treatment what she wanted for her child, not fancy investigations. She did not accept that there was no therapy for this condition. The mother’s perseverance, gave me no chance to rest on the strength of a fine diagnosis”*.

[24]

Mary’s persistence triggered Drs. Bickel, Gerrard and Hickmans to look for a treatment for Sheila. Would the removal of phenylalanine from her diet be a possible treatment? It was only a theoretical possibility, and Sheila was discharged after just over two weeks in hospital. However, Mary’s persistence had paid off, although she would have to wait another seven weeks while the doctors set about their plans to make a low-phenylalanine diet for her daughter.

## 5. Dietary Treatment of PKU—The Prototype

The idea of a diet as a possible treatment for PKU was not new, but discussions in the 1930s had questioned whether the presumed biochemical abnormality was the cause of the clinical features in PKU, and whether a nutritionally adequate diet could be produced if phenylalanine was removed [25,26]. In the early 1950s, Dr. Louis Woolf, a chemist at Great Ormond Street Hospital, London [7,27], tried supplementing the diet of two infants with PKU with glutamic acid, hoping to reduce their blood phenylalanine concentrations by increasing its excretion. Blood phenylalanine was not lowered and there was no benefit to the children’s development. Dr. Woolf went on to suggest that it might be possible to prepare a low-phenylalanine diet from casein hydrolysate, by selectively removing the phenylalanine by adsorption chromatography with charcoal. Such a chemical method had been used in the USA in work unrelated to PKU [28,29].

**Figure 5:**
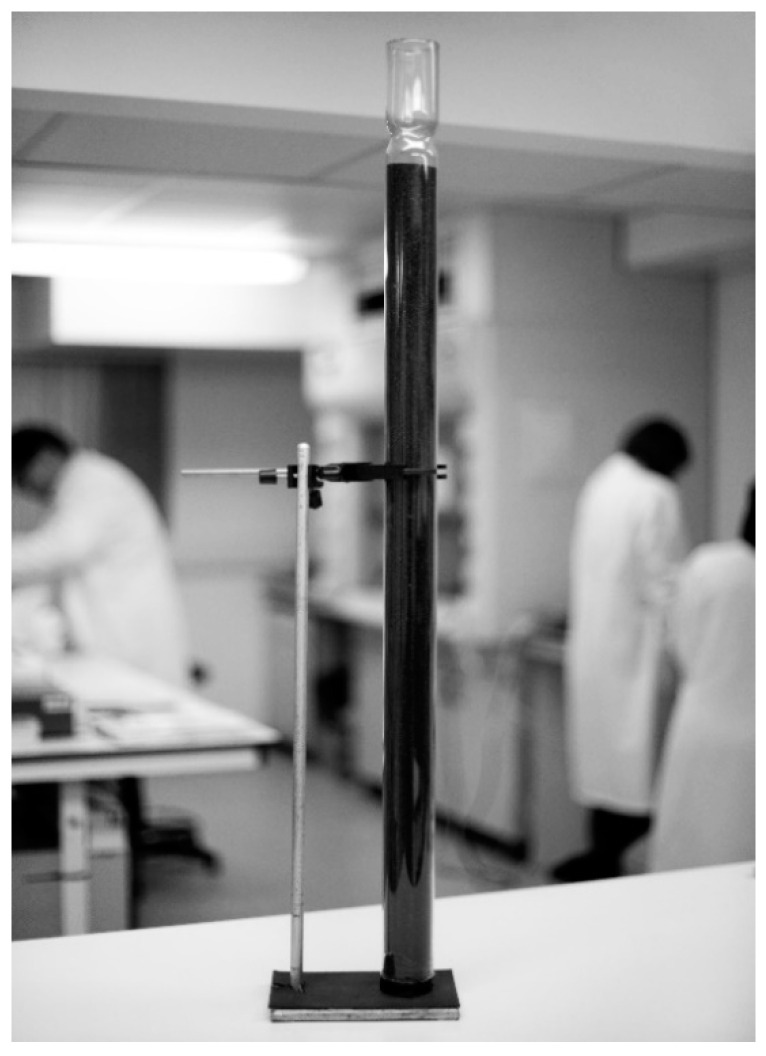
The original glass column filled with charcoal used to prepare phenylalanine-free casein hydrolysate for Sheila’s diet.

Drs. Bickel and Hickmans were aware of Dr. Woolf’s experience and expertise, and he generously shared his ideas with them [6]. Casein hydrolysate was made available to them by the British pharmaceutical company, Allen and Hanburys, and the removal of the phenylalanine was begun by repeatedly mixing a 10% solution of it in acetic acid with acidified charcoal and stirring the mixture in a glass beaker for an hour. The resulting liquid was then passed several times through a glass column containing more charcoal to complete the removal of the phenylalanine, as checked by microbiological assay. The original glass column used in 1951 was 3 ft long × 2 ins diameter (91.5 cm × 5 cm) (Figure 5) and is on display in the Biochemistry Laboratory at Birmingham Children’s Hospital [10] (p. 141).

Amino acid chromatography showed that the charcoal process also removed tyrosine, tryptophan and cystine, but it produced an amino acid mixture that could be used as the basis for the prototype diet that was to be fed to Sheila. It was not an easy procedure to undertake and maintain. It was difficult and time-consuming, and had to be performed in a cold room, and the charcoal got on everything. The image of Dr. Bickel recalled by his colleagues of the time is of him wrapped in layers of sweaters topped by a charcoal- smudged lab coat. Dr. Bickel himself describes how *“He and Dr. Hickmans became as black as coal miners as they prepared the formula”*, and how he worked in the freezing laboratory whilst his family enjoyed a warm Christmas celebration without him [10] (p. 150), [30] (p. 23). After several weeks, enough of the phenylalanine-free product had been prepared, and Sheila was readmitted to Birmingham Children’s Hospital on 21 November 1951. It would be a long stay in hospital for Sheila, with all the disruptive consequences for Mary and her family [10] (p. 42). It was a huge commitment from the doctors and Mary as together they embarked upon what would become a ground-breaking step.

Sheila’s experimental treatment with a phenylalanine-free diet commenced 27 November 1951. After five days, Sheila’s plasma tyrosine levels, which had been low to start with, were no longer measurable. There was great concern as Sheila rapidly lost weight. Pure l-tyrosine was added as a supplement to the diet, and this resulted in a prompt increase of blood tyrosine levels and a temporary halt to her weight loss. After three weeks, the diet (with added tyrosine) was having the effect on Sheila’s blood biochemistry that all had hoped, but she was still losing weight and had become unwell. She developed a generalised aminoaciduria which suggested that she was breaking down her own body protein. On 18 December, 0.8 g phenylalanine in the form of 500mL natural milk was added to her diet, and slowly, over many weeks, Sheila began to regain her lost weight. Improvement in mental condition, however, could not be concluded. Dr. Bickel wrote [31]:

“The assessment of any changes in mental capacity and behaviour of the patient presented great difficulties. The child was too young and of too low intelligence grade to be given any intelligence test. She behaved rather like a nine months old baby. There certainly was no sudden improvement under phenylalanine free food, apart for the fact that she soon became quieter and more contented and ceased banging her head on the pillow. This however might have been because she was now accustomed to her new environment. Livelier interest in her surroundings, a more intelligent expression in her eyes, attempts to stand, etc. were recorded by the nursing staff but are too vague to be indicative of any real improvement.”

Sheila continued on the diet in hospital until March 1952. Her weight began to increase, and on 17 March 1952, she went home. Mary was asked to bring her back to the hospital every week to collect a supply of the diet mixture and for Sheila to see the doctor and have blood tests [10] (p. 46). Further modifications were made to the diet, with reductions in the amount of natural milk and a consequent increase of the phenylalanine-free hydrolysate. The carbohydrate source of oatmeal or rice was changed to gluten-free wheat flour (wheat starch). Some fruit and vegetables were allowed, and sugar was added to make it more palatable. It was still a very unappetizing diet [10] (p. 65).

Over the next six months, an improvement in Sheila’s demeanour was observed; her eyes were brighter, she made eye contact, she smiled, stopped drooling and became more interested in her surroundings. She learnt to crawl, to stand, to push chairs and to climb on to them. Her hair began to thicken and shine and change from fair/blonde to dark brown. Roughness and eczema of the skin disappeared as did the musty, mouse-like smell [9]. Mary, too, noted Sheila’s improvement and Dr. Bickel recorded [31]:

“Since Sheila returned home from hospital her eyes seem brighter and livelier than before. She plays more with toys, crawls more and tried to pull herself up. She makes noises as if she wants to talk. She begins to notice when her name is called whereas before she seemed deaf. She is interested in all food, crawls to pick up a biscuit from the floor and puts it into her mouth. This is the first time she has done this. She has nearly stopped rolling her head from side to side. She has now begun to quarrel with another baby about toys.”

In October 1952, Sheila was three-years-old and had been on the diet for 10 months. She was livelier and played more, but maybe the changes would have happened anyway. Perhaps they were only the result of the extra attention she was receiving. It could not be concluded that the dietary treatment had been responsible for them. Some medical colleagues doubted, not unreasonably, whether it was possible to objectively evaluate the developmental progress in such a young child over such a short time frame. There was also the practical question of continuing to make sufficient quantities of the diet. Its production was time-consuming, and unrelenting with a little girl dependent on it for her food. It was a commitment for Drs. Bickel and Hickmans, and the laboratory, that had to be justified, especially as increasing quantities of the mixture would be required as Sheila grew.

## 6. The Phenylalanine Challenge

The decision to continue treating Sheila with the diet depended upon demonstrating whether or not it had any benefit to her. It had so far been an uncontrolled trial of one. To answer this question, the medical team took a very bold step. They introduced an additional 5 g of pure l-phenylalanine to each day’s supply of the phenylalanine-free casein hydrolysate, in order to mimic the amount Sheila would receive on a normal diet. They decided to do this without Sheila’s mother knowing, thereby avoiding bias in her opinion.

On 9 October 1952, Mary collected Sheila’s weekly supply of the mixture as usual. A few days later she returned to the hospital in tears, and for the first time, she came without Sheila. She stated:

“Within 6 h of starting her ‘food’, Sheila began to cry and roll and bang her head as she used to before being given the special diet. On the following day she had cried continually, her eyes had become heavy and she could no longer stand and scarcely crawl.”

These were dramatic descriptions of changes in Sheila’s condition which had started immediately after the extra phenylalanine had been added [9]. A visit by the doctors to Sheila at home six days later confirmed Mary’s report, with supportive observations by the lodgers. Sheila was promptly put back on the phenylalanine-reduced diet. She was seen in clinic on 23 October when she seemed to be back to her previously, phenylalanine-restricted self.

**Figure 6:**
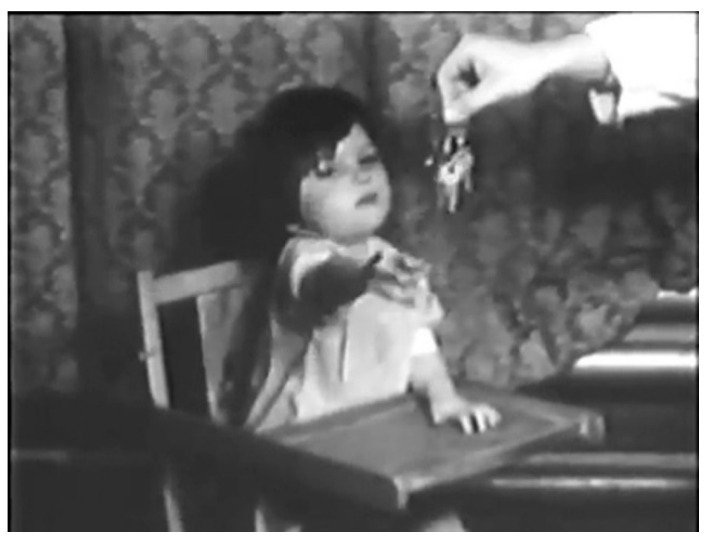
Sheila reaching for Dr. Bickel’s keys, 1952 in Birmingham (photograph reproduced with kind permission of the Jones family).

Sheila was admitted to BCH for the third time on 5 November 1952. She was back on the low-phenylalanine diet and was observed for a period of time. Dr. Bickel recorded her on film [32], alert, playing and interested, although still obviously mentally impaired. She walked with support and laughed when tickled. In the film, Dr. Bickel uses his keys to attract her attention (Figure 6).

On 26 November 1952, this time with Mary’s permission, 4 g l-phenylalanine was added to Sheila’s diet, and the following day, 5 g added. The changes in her behaviour were dramatic. Within 24 h, she became irritable, drowsy and vomited. Her face and eyes became vacant, and she lost interest in her food and surroundings. She developed facial eczema and began to salivate. After 6 days with the added phenylalanine, she could no longer stand or crawl and was not interested in toys or Dr. Bickel’s keys. On 3 December, she was returned to the phenylalanine-reduced diet. Some weeks later, she was able to walk again with support of a chair, she was a lot more interested in playing and was able to crawl onto a chair [32].

It was concluded that excess phenylalanine, or its breakdown products, produced a deleterious effect on mental function in PKU patients [9]. In an addendum to these findings, Dr. Bickel was also able to report [9,33] no untoward reaction in a five-month-old child without PKU who had been given 4g l-phenylalanine for four days. The rise in blood phenylalanine was less (×15) than that observed in PKU patients, so, although not directly comparable, the findings did imply that the deleterious effect of added dietary phenylalanine was specific to PKU patients, and reflected the susceptibility of the brain to the very high blood phenylalanine concentrations found in that condition.

## 7. Further Development

Galvanised by the findings from Sheila, others began dietary treatment for PKU. Dr. Louis Woolf repeated the work in three children at Great Ormond Street Hospital London, using more objective psychometric and EEG measurements [34]. Marked intellectual improvement followed in the two younger children, and in one case, the EEG became normal. Armstrong and Tyler [35] in the USA reported treatment of five PKU cases with a low-phenylalanine diet comprising a mixture of pure l- amino acids as opposed to modified casein hydrolysate. They reported less conclusive evidence of clinical benefit, although blood biochemistry was normalised. Five more infants with PKU began treatment at Birmingham Children’s Hospital under the care of various consultant paediatricians, Dr. Gerrard, Dr. Otto Woolf, and Professor Smellie. Detailed metabolic studies and formal testing of IQ and development were undertaken, and improvements in mental age and IQ were reported in 1956, adding to the evidence of benefit from a low-phenylalanine diet in PKU [36].

By 1954, the phenylalanine-free casein hydrolysate originally prepared in the BCH laboratory for Sheila became commercially available in powder form from Allen and Hanburys [10] (p. 86). This was a very rapid response from industry, a clear recognition of the importance of the Birmingham findings and the need for such a product.

Dr. Hickmans retired from Birmingham Children’s Hospital in 1953. Dr. Bickel, having completed his Ph.D., returned to Germany in 1954 to continue his career in Paediatrics in Marburg and Dr. Gerrard left in 1955 to become head of Paediatrics in Saskatchewan, Canada.

## 8. Sheila 1952–1958

Following her dietary treatment between December 1951 and October 1952, and the phenylalanine challenge in late 1952, Sheila went home on the diet. The schedule of Sheila’s visits to Birmingham Children’s Hospital resumed with more formalised developmental assessments which are detailed in her story [10] (p. 56), [36].

By January 1954, at age 4 years 3 months and after two years of treatment, Sheila was able to stand unaided and could walk a few steps with help. Her attention span was markedly increased and apart from language, which continued to lag markedly, she was assessed overall as having a developmental age of 12 months. In 1954, there was further film evidence of her progress [32] and it was assessed that she had gained around six to seven months in development during the period of being on a phenylalanine-reduced diet.

A difficulty for the doctors had been trying to assess the change in Sheila’s development in response to her low-phenylalanine diet. The lack of objective criteria for the assessment of development in young children over time had hampered them in convincing both themselves and others of the beneficial effects of their experimental therapy. Subsequently, the availability and use of newly developed criteria by Griffiths in 1954 [34] became a critical tool to assess developmental progress as more children were treated and so it was in February 1955, at 5 years 4 months, that Sheila was assessed for the first time using the Griffiths scale [10] (p. 58) This assessment showed that her motor development had further improved but language continued to lag. Overall progress had continued but at a slower rate than the preceding 12 months. Difficulties began to arise in keeping Sheila on her phenylalanine-restricted diet; she was growing and was stronger, and the mixture was extremely unpleasant. Mary persisted in trying to feed it to her until November 1955, but the effort was unsustainable. The special low-phenylalanine hydrolysate mixture was discontinued in December 1955 and restriction of Sheila’s phenylalanine intake was reduced to limiting her natural protein intake. At age 6 years 2 months, Sheila’s loss of dietary control was reflected in her manner; she was more easily distracted and test materials for the developmental assessments were thrown on the floor. Although some further progress was recorded, mental ability was judged to have progressed at a considerably slower rate than previously. At her last assessment in April 1956, when she was 6 years and 6 months, she was judged to be functioning at a mental age of around 18 months.

The break in Sheila’s clinical record at this time coincides with the family’s move to the north of the city. Sheila was almost seven-years-old but with the skills of a two-year-old. Mary had three sons as well as Sheila; her financial resources were next to non-existent; she was on her own, looking after her children, living in a back-to-back house; and the social services of the day provided very limited help. Mary worried about the future for Sheila. Would she ever be able to speak? Would she ever be able to look after herself? Feeding Sheila her special diet was no longer possible, and perhaps Mary finally realised there was no longer any hope that Sheila would get better. Despite these difficulties, she continued to look after Sheila at home in Aston for the next two years.

## 9. Sheila at Chelmsley and Brooklands Hospitals

In September 1958 and again in February 1959, whilst Mary was in Highcroft Hospital, Sheila was admitted to Chelmsley Hospital (a large mental deficiency hospital) near Birmingham for short-term care. The family’s circumstances had not improved. Mary now had a four-week-old baby in addition to her other children, and they were still living in the impoverished back-to-back house in Aston. Mary’s decision to give up looking after Sheila at home was not easy for her but it was inevitable, and so, on 25 July 1959, Mary applied for Sheila to be formally admitted to Chelmsley Hospital under the legislation of the Mental Deficiency Act 1913 as a “mental defective” [10] (p. 127). An appreciation of the difficulties in caring for Sheila is revealed in her clinical notes at the time of her admission: [10] (p. 68).

Sheila continued to be cared for at Chelmsley, apart from a brief visit home in 1963, until the mid-1990s when she transferred to Brooklands Hospital, as part of the reorganisation of the Mental Health Services in the UK [37]. For over 20 years Mary had continued to regularly visit her daughter at Chelmsley until her own illness and death in 1981. For Sheila, Brooklands provided a non-institutionalised, “homely” environment where she lived a semi-domestic life with four or five other patients in bungalow accommodation [10] (p. 81) until she died, aged 49 years on 29 January 1999.

Birmingham Children’s Hospital were not aware that Sheila was being cared for at Chelmsley until 1987 when she was investigated for PKU [10] (p. 80). At that time, the BCH metabolic team rediscovered Sheila who was functioning with severe learning disability. It was a sad and a stark reminder, particularly to those involved with newborn screening, of the meaning and reality of untreated PKU [10] (p. 81) and what the future meant for those with PKU prior to newborn screening.

## 10. The Legacy

During the 40 years that Sheila was being cared for at Chelmsley/Brooklands, much had happened to better understand, diagnose and treat PKU. The success of the laboratory-made protein substitute used to treat Sheila provided the stimulus for the commercial manufacture of similar products. Within two years, Allen and Hanburys were marketing “Cymogran” following the original formula prepared in Birmingham. Another British firm, Trufood, developed “Minafen” specifically for infants, based on the work by Louis Woolf in 1955 [34]. Two products were developed in America: “Lofenalac” by Mead Johnson in 1958 and “Ketonil” by Merck, Sharp and Dohme. In 1960, another protein substitute, “Albumaid XP”, using hydrolysed albumin as the source of amino acids, was produced by Scientific Hospital Supplies Ltd. from Liverpool UK. The availability of such products enabled other hospitals to begin treating patients, and services for management of those with PKU developed at a rapid pace [10] (p. 88). Further developments of the dietary products for PKU have continued with greater understanding of the nutritional requirements and efficacy [38].

By the time Sheila was admitted to Chelmsley, more infants with PKU were being treated providing greater evidence of the benefits of the diet [39]. In 1960, analysis of data on 55 cases from England and the USA showed that the number of children who attain an IQ of over 60% declines steeply if the age of starting treatment is over one year, whereas almost all those treated in the first few weeks of life have an IQ within the normal range [40]. Additionally, in 1960, Dr. Bickel, now working in Marburg, reviewed the treatment of a further 10 patients and 79 cases reported by others [41]. The benefits to mental development of diagnosing patients soon after birth and beginning treatment early were becoming clearer, and so the need for screening programmes for PKU in the newborn period was championed as a priority. Throughout the late 1950s and 1960s, whilst newborn screening programmes were being introduced, the role of the family doctor and paediatrician was emphasised by encouraging testing of any baby or infant with signs and symptoms suggestive of PKU, such as eczema, failure to thrive, febrile convulsions, and slow development, to enable the earliest possible diagnosis and chance of preventing severe brain damage.

The first screening programmes in the UK used liquid urine [42]. In 1959, Birmingham pioneered newborn screening using *Phenistix* (the “nappy” test) with testing of all babies at six weeks of age, the same year Sheila was admitted to Chelmsley [43]. Urine testing was replaced by the more reliable blood-based screening method for phenylalanine, developed by Robert Guthrie in the USA [44], the now familiar “heel-prick”, or “blood spot test”. In the UK, many locally organised programmes quickly followed, and by the early 1960s, newborn screening for PKU was established across most of England and Wales [45,46]. This was only 10 years after Sheila had been treated.

## 11. Conclusions

The creation and implementation of newborn screening programmes for PKU, and the subsequent, successful treatment of affected infants, is a huge success story. In less than 20 years from the time Mary Jones pestered Dr. Bickel wanting treatment for her daughter, newborn screening for PKU had travelled the world. It would not be an overstatement to say that Sheila’s legacy is today’s newborn screening programmes, wherever they are found.

## 12. Discussion

In reviewing Sheila’s legacy, it is worthwhile reflecting on some of the key issues that Mary Jones and the pioneer medical team faced as they made the journey towards a treatment for PKU.

The social circumstances which existed at the time were undoubtedly a factor in why Sheila did not continue on the diet. While statements such as “*Mrs. Jones was no longer able to continue the diet and had abandoned it*” are factually true, they do not reflect the hardships Mary faced during her life. She was a caring mother, but had four sons and a daughter with severe learning disability, and little income. Social services barely existed. The NHS, formed in 1948, was still in its early years and hospitals operated independently from community services. Mental Health Services were separate from both. When Mary moved her family to the then impoverished area of Aston, her contact with the health system became disrupted and Sheila became lost to it. When Sheila was admitted to Chelmsley Hospital, very few involved in medical care at that time, and at any level, were aware of PKU. A single note in Sheila’s records at Chelmsley mentions PKU but ascribes it no significance to her care, leaving her to be rediscovered by the medical system thirty years later.

Challenging Sheila with added phenylalanine in her diet was a key decision in the experimental testing of her treatment, made remarkable because it was initially made without asking her mother. We do not know how Mary Jones reacted when she was informed about what had happened, but we do know she agreed to repeating the experiment in hospital and for Sheila to be filmed. Under current ethical standards, such experimenting would not be permitted today. Sheila was an uncontrolled trial of 1 and had the doctors not had the courage to proceed, she would not have been treated, and the discovery of the treatment for PKU with diet would have been delayed, possibly for decades.

Undoubtedly, serendipity has a place in the story. Prior to 1951, the diagnosis of PKU was infrequent and required specialist doctors who were interested to look for a rare and then untreatable disease. Diagnosis also required a laboratory capable of performing the advanced biochemical analyses necessary to confirm it. This was a rare combination. By chance, it came together in Birmingham in the late 1940s. Even more extraordinary, it embodied opposite sides of the recently ended WWII. It was not usual for a German doctor to secure a post in England at that time, although the fact that Dr. Bickel’s wife was English may have helped to bring it about. Additionally, the fact that the respective wives of Dr. Bickel and Dr. Gerrard already knew each other and Dr. Gerrard had friends in Germany was undoubtedly a help in forming friendships. Dr. Jon Gerrard (son of Professor John Gerrard) reflected on this [10] (p. 102):

*“It was an extraordinary demonstration of how these two individuals were able to collaborate together so soon after the war to make such a major advance in helping children with PKU”*.

(personal communication Jon Gerrard 2019)

The third member of the clinical team, Dr. Evelyn Hickmans, was an inspired scientist who developed and introduced many innovative laboratory techniques and was only the second chemist in England to be employed in a hospital laboratory at that time [18]. The team was thus in place when Mary Jones brought Sheila to Dr. Gerrard’s clinic in March 1951, and what followed was yet another demonstration that, in medical advances, it is the team effort of science, medicine and family together with courage that produces the result.

## 13. Recognition

**Figure 7:**
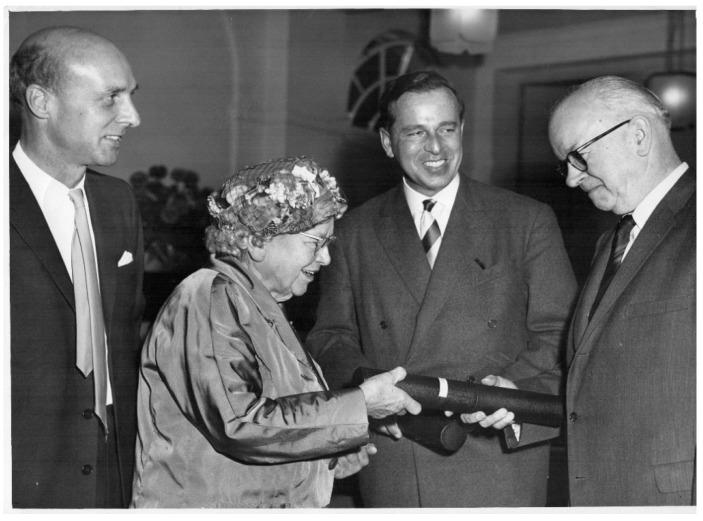
Dr. Evelyn Hickmans with Professor John Gerrard (left) and Professor Horst Bickel (right) receiving the John Scott Award from Dr. W Greulich, Scientific attaché to the US Embassy in London: Birmingham 1962.

The demonstration that PKU could be treated with diet and the severe brain damage prevented led to the professional recognition of those involved. Dr. Følling, who first described the metabolic basis of PKU, was awarded the Joseph F Kennedy International Award in Mental Retardation in 1962 [31] (p. 21), [47]. In the same year, Drs. Bickel, Gerrard and Hickmans were awarded the John Scott Award for the discovery of the dietary treatment [48,49] (Figure 7). In 2019, the International Society for Neonatal Screening awarded Dr. Louis Woolf a plaque to recognize his contribution [5,7,50].

Only lately has Sheila’s contribution been formally recognised. In 2017, the Sheila Jones Award was created by the European Society for PKU, with the first award presented to Laura Petreus of Romania [51]. This annual award is for advocacy in the cause of individuals with PKU, the characteristic so determinedly displayed by Mary Jones when she demanded and then persisted with treatment for her daughter. For those families living with PKU it is, perhaps, the most important award of all.
